# Integrated physiological, metabolomic, and transcriptomic analyses elucidate the regulation mechanisms of lignin synthesis under osmotic stress in alfalfa leaf (*Medicago sativa* L.)

**DOI:** 10.1186/s12864-024-10039-1

**Published:** 2024-02-13

**Authors:** Jing Yang, Jiangnan Yi, Shihai Ma, Yafang Wang, Jiaxing Song, Shuo Li, Yueyan Feng, Haoyang Sun, Cai Gao, Rongchen Yang, Zhongxing Li, Yuman Cao, Peizhi Yang

**Affiliations:** College of Grassland Agriculture, Northwest A&F University, 712100 Yangling, China

**Keywords:** Alfalfa, Lignin, Transcriptome, Metabolome, Osmotic stress

## Abstract

Alfalfa, an essential forage crop known for its high yield, nutritional value, and strong adaptability, has been widely cultivated worldwide. The yield and quality of alfalfa are frequently jeopardized due to environmental degradation. Lignin, a constituent of the cell wall, enhances plant resistance to abiotic stress, which often causes osmotic stress in plant cells. However, how lignin responds to osmotic stress in leaves remains unclear. This study explored the effects of osmotic stress on lignin accumulation and the contents of intermediate metabolites involved in lignin synthesis in alfalfa leaves. Osmotic stress caused an increase in lignin accumulation and the alteration of core enzyme activities and gene expression in the phenylpropanoid pathway. We identified five hub genes (*CSE*, *CCR*, *CADa*, *CADb*, and *POD*) and thirty edge genes (including *WRKY*s, *MYBs*, and *UBPs*) by integrating transcriptome and metabolome analyses. In addition, ABA and ethylene signaling induced by osmotic stress regulated lignin biosynthesis in a contradictory way. These findings contribute to a new theoretical foundation for the breeding of high-quality and resistant alfalfa varieties.

**Supplementary Information:**

The online version contains supplementary material available at 10.1186/s12864-024-10039-1.

## Introduction

As sessile organisms, plants have to adapt to changing environments. Osmotic stress can be caused by plants subjected to various abiotic stresses, including drought, salt, and low temperature stresses [1]. When plants are threatened by osmotic stress, the cells lose water seriously, which adversely affects the normal growth of plants. Abiotic-induced osmotic stress severely limits plant biomass production and poses significant threats to agricultural industries [2]. Consequently, it is necessary to investigate the response mechanism of plants under osmotic stress. Understanding of how plants respond to osmotic stress is important not only for basic biology but also for agriculture [1]. Some studies have shown that plants can resist adverse environmental stress by increasing lignin deposition [3]. Lignin is an important component of the plant cell wall, which is conducive to the growth, development, and water transport of plants, and it can also alleviate damage to plants by adverse external environments or pathogens [4]. *MeRAV5* improved lignin accumulation to enhance drought stress resistance by promoting the activities of both *MePOD* and *MeCAD15* in cassava [5]. Overexpression of *MdSND1* increased the lignin content in apples and improved the salt and osmotic stress resistance [6]. Overexpressing of *AgNAC1* enhanced the plants’ resistance to salt stress by increasing lignin accumulation in *Arabidopsis* [7]. Although lignin is beneficial to plant resistance to osmotic stress, knowledge of lignin response to osmotic stress is still limited.

Previous studies based on the model plant *Arabidopsis thaliana* and the model woody plant poplar have identified 11 core enzymes related to lignin biosynthesis in plants [8, 9]. Lignin synthesis was affected by enzyme activities and the expression levels of the genes that encode these enzymes. L-phenylalanine ammonia-lyase (PAL), cinnamate 4-hydroxylase (C4H), and 4-hydroxycinnamate CoA ligase (4CL) are the three common enzymes that belong to the general phenylpropanoid pathway. The other eight enzymes, including caffeoyl shikimate esterase (CSE), hydroxycinnamoyl CoA: shikimate hydroxycinnamoyl transferase (HCT), coumarate 3-hydroxylase (C3H), ferulate 5-hydroxylase (F5H), caffeic acid O-methyltransferase (COMT), caffeoyl CoA 3-O-methyltransferase (CCoAOMT), cinnamyl alcohol dehydrogenase (CAD) and cinnamoyl CoA reductase (CCR), belong to lignin-specific pathway [10]. In addition, some transcriptional factors (*NAC* and *MYB*) are important to lignin synthesis [11]. Some of the *NAC* transcription factors were regarded as the switch of lignin synthesis by regulating the genes involved in cell wall biosynthesis in *Arabidopsis* [12]. Transcriptional activation of *OsCCR10* by *OsNAC5* enhanced the ability to resist drought stress by increasing lignin content in the root of rice [13]. Except for the *NAC* genes family, *MYB* transcription factors were extensively related to the transcriptional regulation of lignin. *MYB58* and *MYB63* positively regulated cell wall formation by lignin synthesis in *Arabidopsis* [14]. As a transcriptional activator of the lignin synthesis of populus, *PtoMYB92* contributed to cell wall formation [15]. *CmMYB15* regulated the biosynthesis of lignin in chrysanthemum [16]. *CsMYB330* and *CsMYB308* promoted fruit lignification by regulated expression of the *Cs4CL1* gene in *Citrus sinensis* [17]. The synthesis and regulation of lignin have been studied in several plants, but how osmotic stress regulates lignin synthesis has not been systematically investigated.

Alfalfa is an important leguminous forage with high nutritional value and strong adaptability, and it is an autotetraploid with a genome size of 2.738 Gb and 32 chromosomes, including eight homologous groups with four allelic chromosomes in each [18]. In China, alfalfa grows mostly in arid and semi-arid areas and often suffers from osmotic stress caused by drought or salt stress, which threatens the yield and quality of alfalfa. Although lignin can improve the ability of alfalfa to adapt to abiotic stress, it can also reduce the forage digestibility. Therefore, exploring the synthesis and regulation mechanism of lignin under osmotic stress is important. Previous studies have shown that an increase in l-phenylalanine content provides favorable conditions for lignin synthesis in alfalfa leaves, which is one of the main factors contributing to a decline in alfalfa RFV and quality [19]. However, how osmotic stress regulates lignin biosynthesis in leaves is unclear. The assimilates were synthesized in mesophyll cells through photosynthesis and then transported through the vascular systems to stems for growth [20] and structural carbohydrate (lignin and cellulose) biosynthesis [21]. The lignification of leaf vasculature affected water and nutrient transport and lignin accumulation in the stems. In this study, we explored the lignin synthesis mechanism in alfalfa leaves under osmotic stress by combining physiological, metabolomic, and transcriptomic analyses. We investigated the activities of key enzymes related to lignin biosynthesis as well as metabolite accumulation levels, identified key structural genes of lignin in response to osmotic stress, and constructed a gene co-expression regulatory network. We conducted combined metabolomic and transcriptomic analyses to identify genes involved in lignin biosynthesis under osmotic stress, providing a theoretical basis for the selection of target genes in the molecular breeding of alfalfa. Our findings provide new insights for breeding high-quality or improved-stress-tolerant alfalfa varieties.

## Results

### Lignin accumulation of alfalfa under osmotic stress

**Fig. 1:**
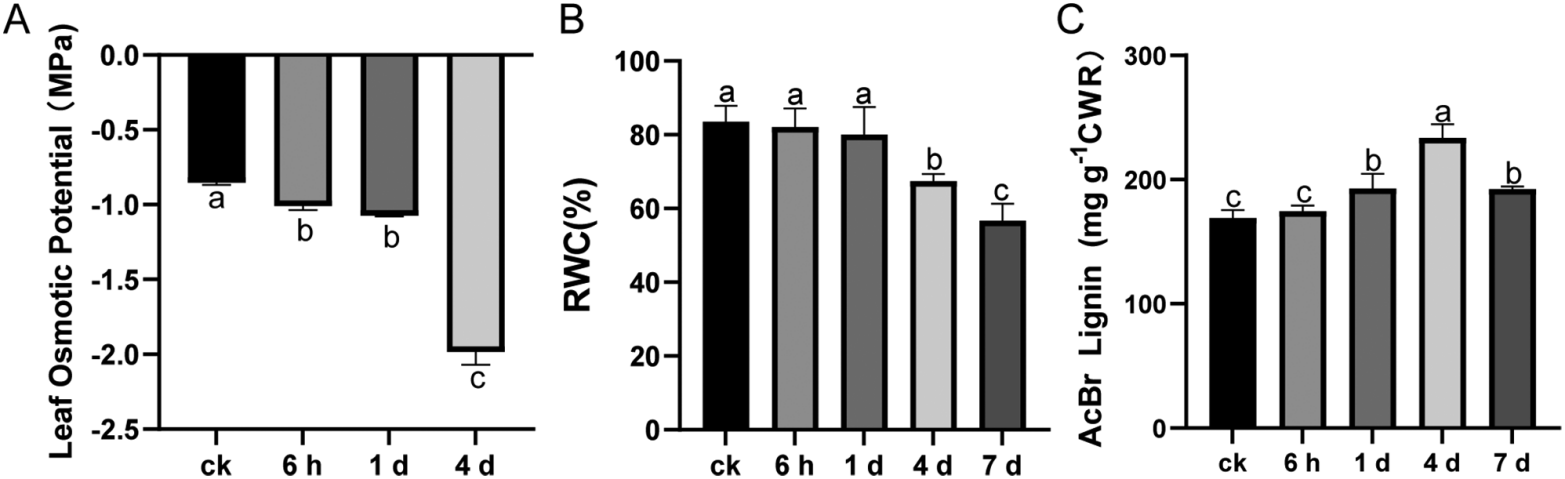
The changes of osmotic potential (**A**), relative water content (**B**), and lignin content (**C**) in the leaf tissue of alfalfa under osmotic stress. Data were presented as the mean standard deviation (SD). Different letters (**a**, **b**, and **c**) indicate significant differences (*P* < 0.05)

To investigate the changes in alfalfa lignin accumulation under osmotic stress, hydroponics alfalfa was treated with 100 mM mannitol for 6 h, 1 d, 4 d, and 7 d. The osmotic potential of alfalfa leaves decreased significantly (*P* < 0.05) on the 6 h and 1 d of osmotic stress and continued to decrease greatly on the 4 d (Fig. 1A). The relative water content (RWC) of alfalfa leaves decreased significantly (*P* < 0.05) on the 4 d of osmotic stress and reached the lowest level on the 7 d (Fig. 1B). The lignin content of alfalfa leaves increased significantly (*P* < 0.05) from 1 d to 4 d of osmotic stress, and decreased on 7 d, which was higher than the normal growth conditions before mannitol treatment (CK) (Fig. 1C). These results indicated that osmotic stress significantly raised the content of lignin in leaves of alfalfa.

### Analyses of metabolome accumulation and enzyme activity of lignin biosynthesis pathway in alfalfa leaves

**Fig. 2:**
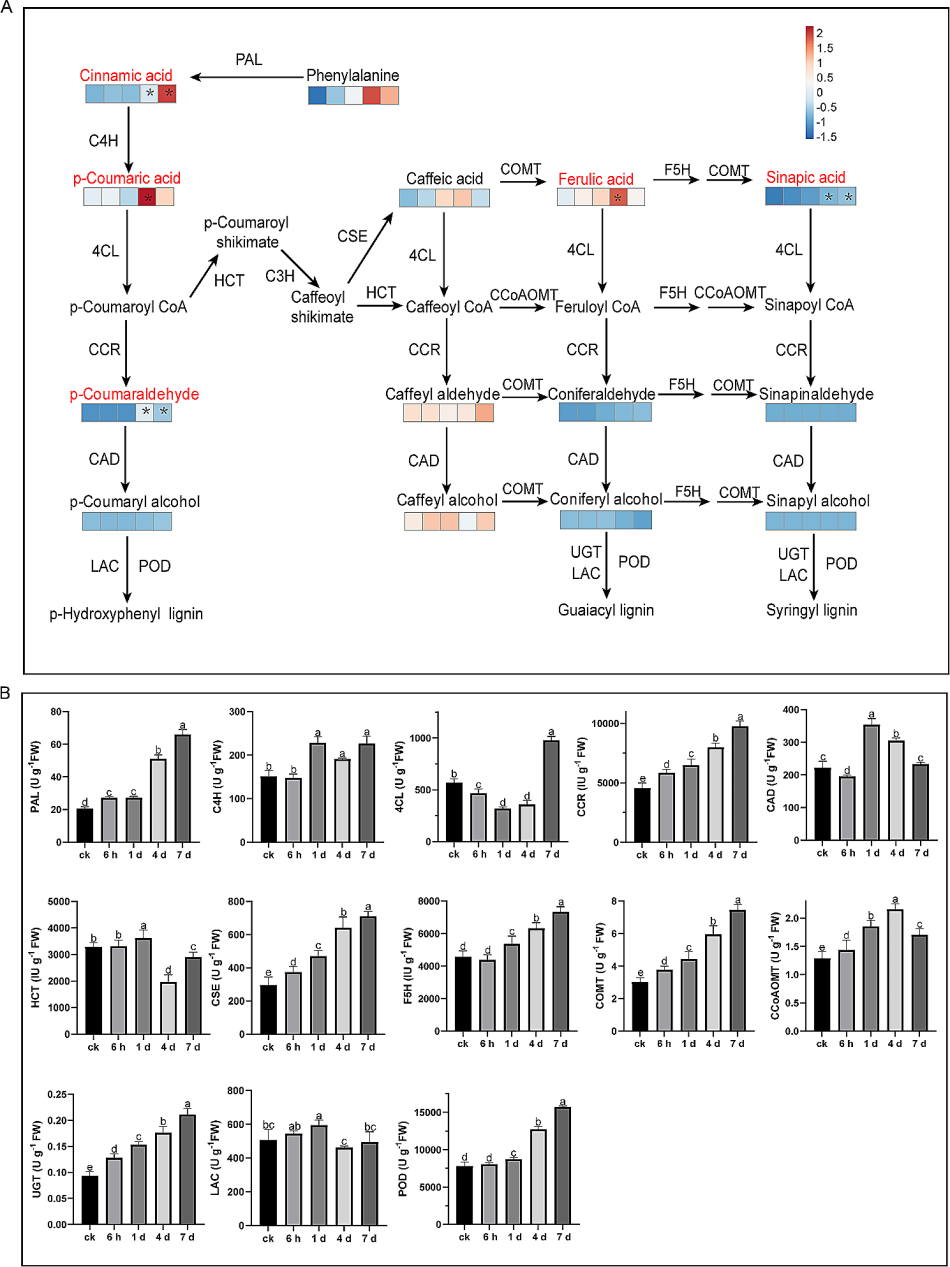
The metabolite contents (A) and enzyme activities (B) related to lignin synthesis under osmotic stress in the leaves of alfalfa. PAL, phenylalanine ammonia-lyase; 4CL, 4–coumaric acid: CoA ligase; C4H, cinnamate 4-hydroxylase; CCR, cinnamoyl-CoA reductase; HCT, hydroxycinnamoyltransferase; CSE, caffeoyl shikimate esterase; F5H, ferulate5-hydroxylase; CCoAOMT, caffeoyl-CoA 3-O-methyltransferase; COMT, caffeic acid O-methyltransferase; UGT, uridine diphosphate glycosyltransferases; CAD, cinnamyl alcohol dehydrogenase; LAC, laccase; POD, peroxidase. The asterisk represents DAMs with a|log2-fold change| > 1 and P < 0.05. Different letters indicate significant differences (P < 0.05)

The content changes of the metabolites related to lignin synthesis were analyzed in alfalfa leaves (Fig. 2A). The ion intensity of metabolite was compared between the different periods of osmotic stress and the CK conditions to investigate the differentially accumulated metabolites (DAM). Among the 14 identified metabolites, five increased significantly (*P* < 0.05) under osmotic stress and others maintained a relatively stable level. Contents of cinnamic acid, sinapic acid, and *p-coumaraldehyde* increased significantly after 4 d and 7 d, while *p-coumaric* acid and ferulic acid contents significantly increased (*P* < 0.05) on the 4 d and then decreased after osmotic stress for 7 d. These results implied that intermediate metabolites related to lignin biosynthesis accumulated after long-term (4 days and 7 days) osmotic stress in the leaf.

We further determined the activities of enzymes related to lignin biosynthesis. The activities of PAL, CSE, CCR, UGT, and COMT, continuously increased from 6 h to 7 d of osmotic stress compared with CK (Fig. 2B), whereas the activities of C4H, F5H, and POD increased from 1 d to 7 d. The activities of HCT, CCoAOMT, and LAC increased and then decreased under osmotic stress. Although the activity of CCoAOMT was decreased on 7 d, the level of CCoAOMT activity was significantly higher after 7 d of osmotic stress compared with CK. Interestingly, the activity of 4CL decreased from 6 h to 4 d of osmotic stress and then increased greatly on 7 d. The CAD activity fluctuated during osmotic stress, which reached the peak on 1 day of osmotic stress and maintained the same level as CK on 7 d (Fig. 2B). Osmotic stress induced dynamic changes of enzyme activities related to lignin biosynthesis, and the majority of these enzymes showed increased activities under osmotic stress.

### Transcriptome profile of alfalfa under osmotic stress

**Fig. 3:**
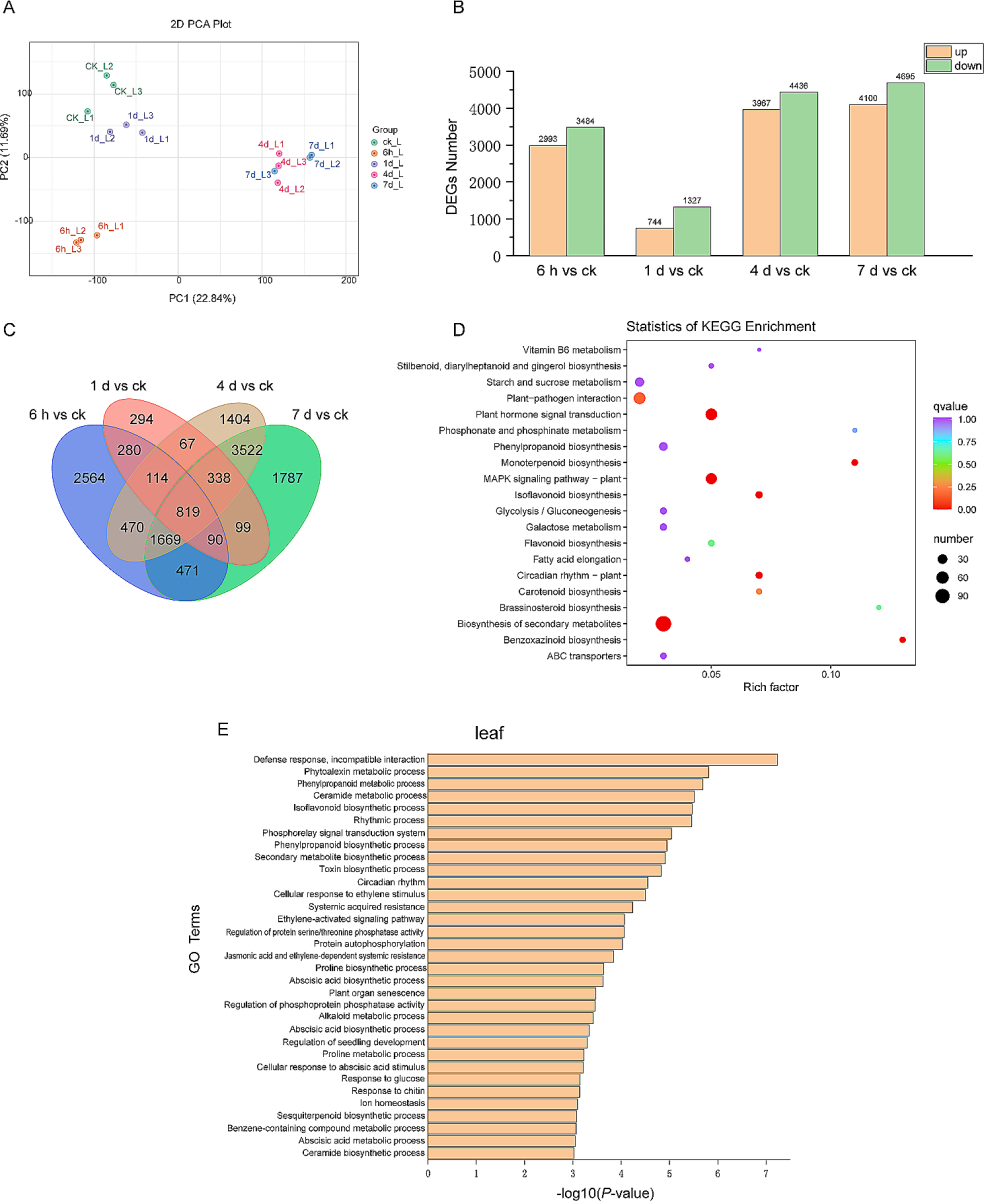
Transcriptome analysis between osmotic stress treatment and normal growth conditions (ck) in alfalfa leaves. (A) Principal component analysis of gene expression. (B) The number of differentially expressed genes (DEGs) for 4 groups of comparison (6 h vs. ck, 1 d vs. ck, 4 d vs. ck, 7 d vs. ck) (C) Venn diagram of the DEGs shared by different times of osmotic stress in leaves. Kyoto Encyclopedia of Genes and Genomes (D) and Gene Ontology (E) enrichment analysis of the common DEGs

To investigate the molecular response of alfalfa under osmotic stress, we compared the transcriptome profile of leaves between different periods of osmotic stress and CK conditions. In the result of principle component analysis (PCA), the samples were clustered into two groups by PC1, suggesting that gene expression patterns were distinct between short-term (CK, 6 h, and 1 d) and long-term (4 days and 7 days) osmotic stress (Fig. 3A). Interestingly, the mannitol treatment for 6 h was separated from CK and long-term stress by PC2, implying that application of mannitol for 6 h might induce shock stress in alfalfa leaves.

A total of 13,988 DEGs were performed with a *P* value < 0.05 and|log_2_-fold change| > 1. Under osmotic stress, the number of DEGs on 1 d was the least, while the DEGs on 6 h, 4 days, and 7 days gradually increased (Fig. 3B). As shown in the Venn diagrams, there were 819 DEGs in different osmotic stress periods (Fig. 3C). The 819 common DEGs were performed with GO and KEGG analysis. The KEGG analysis showed that the most enriched pathways were plant hormone signal transduction, MAPK signaling, and biosynthesis of secondary metabolites (Fig. 3D). These DEGs were enriched into 57 GO terms with a threshold of -log_10_ (*p*-value) > 3 (Table S2). The most enriched biological process (BP) terms were defense response, phytoalexin biosynthesis, phenylpropanoid metabolic and biosynthesis processes, secondary metabolite biosynthesis, cellular response to ethylene stimulus, ethylene-activated signaling pathway, abscisic acid-activated signaling pathway, and abscisic acid biosynthesis (Fig. 3E). These results indicated that phytohormones, especially ABA and ethylene may participate in regulating osmotic-induced lignin synthesis.

**Fig. 4:**
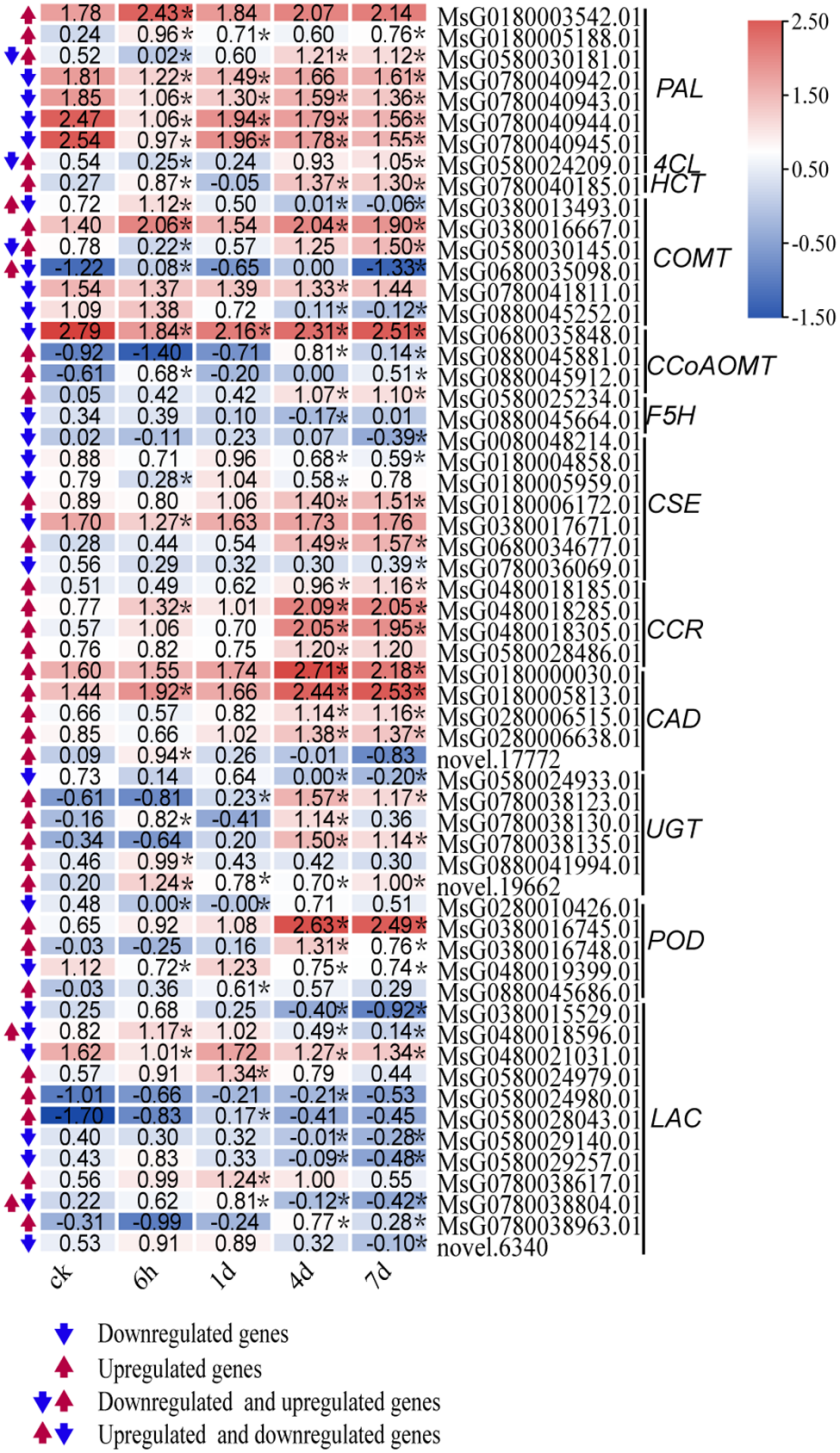
Differentially expressed structural genes identified related to the lignin synthesis in alfalfa leaves under osmotic stress. The red arrows represent upregulation and the blue arrows represent downregulation, respectively. The asterisk indicates DEGs with a|log_2_-fold change| > 1 and *P* < 0.05

We further identified 59 DEGs that encode enzyme proteins directly involved in the lignin synthesis pathway (Fig. 4). Among these DEGs, 31 genes were upregulated in expression, whereas 21 were downregulated by mannitol treatments. All of the *CCR* (4 genes), *CAD* (5 genes), and *HCT* (1 gene) were up-regulated under osmotic stress. Five of the six *UGT* genes showed up-regulated expression levels, and three of the five *POD* genes were up-regulated by mannitol treatment. Two of the *CCoAOMT* genes were up-regulated under osmotic stress. furthermore, five of the seven *CSE* genes were downregulated under osmotic stress. Among the 7 *PAL* genes, four genes were downregulated. The expression level of *4CL* decreased at 6 h of osmotic stress and increased at 7 d. The transcriptional levels of the genes belonging to *COMT*, *F5H*, and *LAC* showed a complex or fluctuated change under osmotic stress. The expression levels of genes related to lignin biosynthesis were differently regulated by osmotic stress. However, more genes were up-regulated by osmotic stress, especially the *CCR*, *CAD*, and *UGT* gene families.

### Co-expression network analysis associated with lignin biosynthesis under osmotic stress

**Fig. 5:**
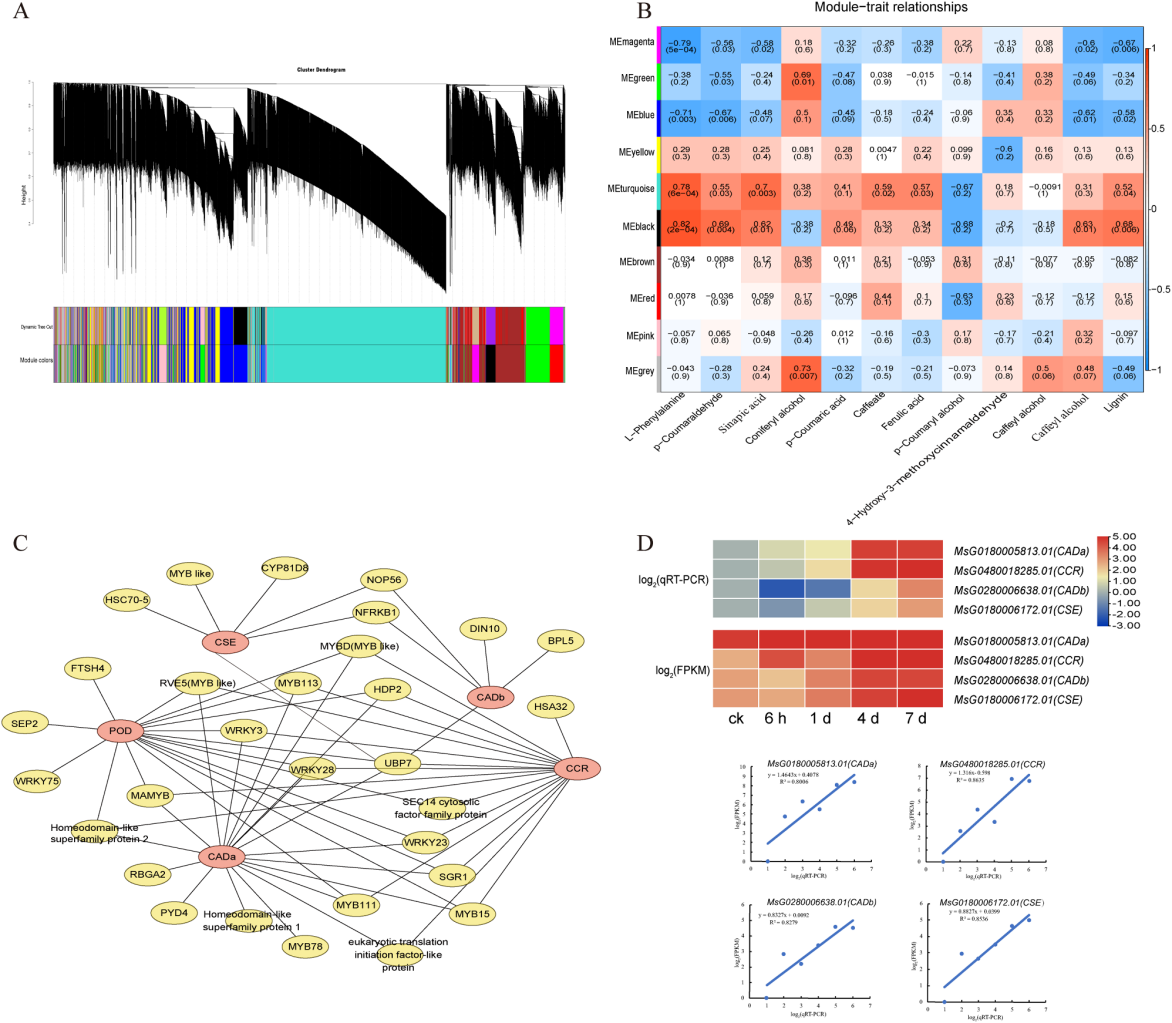
Weighted gene coexpression network analysis (WGCNA). (**A**) Module hierarchical clustering diagram from the WGCNA analysis of alfalfa leaves under osmotic stress. (**B**) The module-trait relationship heatmap. (**C**) Coexpression network of genes involved in lignin synthesis under osmotic stress. The ID and functional annotation of genes were listed in Table S3. (**D**) The relative expression levels of hub genes from RNA-seq and RT-qPCR and their correlation analysis

To investigate the gene co-expression network of lignin synthesis of alfalfa under osmotic stress, WGCNA was analyzed with total genes. We identified 10 modules labeled with different colors, and each module included DEGs with similar expression patterns (Fig. 5A). The module-trait relationships revealed that turquoise module (*r* = 0.52) and black module (*r* = 0.68) were significant modules, which were positively correlated with lignin content and more than 4 metabolites (*r* > 0.55) (Fig. 5B). Among the DEGs belonging to these two modules, five hub genes were identified with top 5 KTotal, including *MsG0180006172.01*(*CSE*), *MsG0480018285.01*(*CCR*), *MsG0180005813.01* (*CADa*), and *MsG0280006638.01*(*CADb*), *MsG0380016745.01*(*POD*) (Fig. 5C). Moreover, thirty edge genes were identified highly correlated with hub genes. These edge genes were mainly belonging to the *MYB* transcription factor, *WRKY* transcription factor, and ubiquitin-specific protease (Fig. 5D). The gene annotation for the edge genes was listed in Table S3. These results suggested that the regulatory networks of lignin biosynthesis were complex and multilayered under osmotic stress. Meanwhile, RT-qPCR was performed to analyze the relative expression of four hub genes to confirm the reliability of the RNA-Seq data. The results showed a high correlation between RT-qPCR and RNA-Seq data (*R*^*2*^ > 0.8) (Fig. 5D).

### Effect of exogenous ABA or ACC treatments on lignin deposition of alfalfa under osmotic stress

**Fig. 6:**
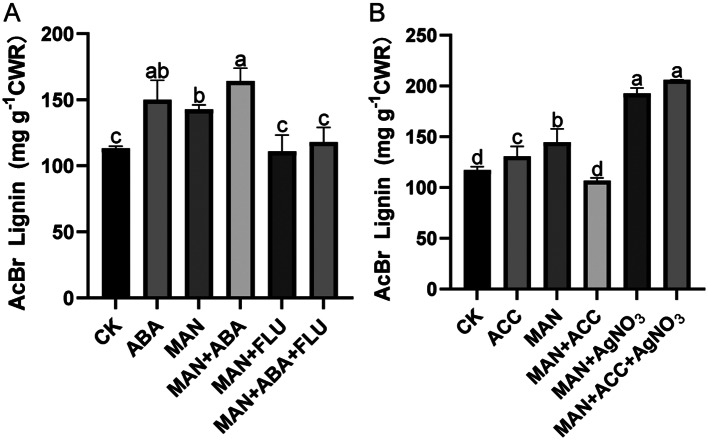
Effect of exogenous ABA or ACC on lignin synthesis in alfalfa leaves. Lignin accumulations in alfalfa leaves under the ABA treatment (**A**) and ACC treatment (**B**). CK, normal growth condition; ABA, abscisic acid; MAN, mannitol; FLU, fluridone; ACC, 1-aminocyclopropane-1-carboxylic acid. Data were presented as the mean ± standard deviation (SD). Different letters indicate significant differences (*P* < 0.05)

GO and KEGG analyses suggested that ABA or ethylene might participate in regulating osmotic stress-induced lignin synthesis. Lignin content was measured by exogenous application of ABA or ACC. As shown in Fig. 6A, ABA or mannitol (MAN) treatments significantly increased lignin content compared with CK (normal growth condition). The combination of ABA and MAN treatment induced more lignin deposition than MAN treatments. The application of fluridone (FLU, ABA inhibitor) significantly reduced lignin accumulation under mannitol treatments. When simultaneously applied with mannitol, ABA, and FLU, alfalfa showed lower lignin content than ABA + MAN, implying that the increased lignin accumulation induced by ABA was alleviated by FLU. These results showed that ABA positively regulated lignin accumulation under MAN-induced osmotic stress.

We analyzed the influence of ethylene on lignin synthesis under osmotic stress and found that lignin content was significantly higher under ACC (ethylene synthesis precursor) treatment than CK (normal growth condition). However, lignin content significantly decreased with ACC and mannitol treatments in comparison with only MAN treatment. These results suggested that the effect of ACC on lignin accumulation was different between CK and MAN-induced osmotic stress (Fig. 6B). The application of AgNO_3_ (ethylene biosynthesis inhibitor) significantly increased lignin accumulation under MAN treatment (Fig. 6B). When simultaneously applied ACC and AgNO_3_ under MAN treatment, alfalfa showed higher lignin content than that of MAN + ACC treatments, implying that the inhibition effect of exogenous ACC on lignin accumulation was alleviated by AgNO_3_ (Fig. 6B). These results shows that ethylene positively regulated lignin accumulation under CK conditions, but negatively regulated the accumulation of lignin under osmotic stress.

## Discussion

As an important component of the cell wall, lignin helps plants to resist adverse external environments, including biotic and abiotic stress. Drought, salt, and other abiotic stresses always cause osmotic stress, and the mechanism of how lignin responds to osmotic stress remains unclear. Here, the combination of physiological, transcriptional, and metabolic analysis was used to reveal the regulation mechanism of alfalfa lignin under mannitol-induced osmotic stress.

In this study, after mannitol-induced osmotic stress for 1 d and 4 d, the lignin content of alfalfa leaves increased significantly. The increase in lignin accumulation might contribute to enhancing osmotic tolerance in alfalfa leaves. The lignin content decreased on 7 d with a higher level than CK. We speculated that mannitol treatment for 7 days may result in severe damage to plant physiological processes as the RWC of alfalfa is below 60%. Alternatively, the photosynthetic products or intermediate metabolites necessary for lignin synthesis were not sufficient for lignin synthesis under long-term osmotic stress. Several previous studies have found that drought or salt stress has a positive effect on lignin synthesis in different plants. The leaf lignin content of maize was increased significantly under severe and moderate drought stress, and lignin was used as an index for the evaluation of drought stress in maize [22]. Similarly, drought stress led to increased lignin accumulation in the primary root of chickpeas [23]. Our study not only suggested that osmotic stress can also regulated positively lignin biosynthesis in alfalfa, but also further elucidates the regulation mechanisms of lignin synthesis under osmotic stress at the level of metabolites and genes transcription expression.

There are many intermediate metabolites involved in the lignin synthesis process. Previous research has pointed out that the content of these intermediate metabolites changes under abiotic stress [24]. Seven DAMs involved in lignin synthesis increased remarkably under salt stress in the root tissue of *Sophora alopecuroides*, including phenylalanine, cinnamic acid, ferulic acid, cinnamaldehyde, caffeylaldehyde, sinapyl alcohol, and coniferin [25]. Under low-temperature stress, the lignin content of tobacco leaves increased, and the accumulation of intermediate metabolites related to lignin synthesis such as p-coumaric acid, p-coumaroyl, and ferulic acid also increased [26]. In this study, we identified 5 DAMs that increased in alfalfa leaves, including cinnamic acid, *p*-Coumaric acid, ferulic acid, *p*-Coumaraldehyde, and sinapic acid. In our previous study, 8 DAMs related to lignin synthesis decreased under osmotic stress due to the dynamic changes in enzyme activities [27]. Comparatively, the increased metabolites in the leaves were also associated with changes in enzyme activities. PAL, C4H, and 4CL are three key enzymes of general metabolic pathways in phenylpropanoid biosynthesis; they catalyze phenylalanine to p-coumaroyl-CoA [28]. The increased accumulation of *p*-Coumaric acid and cinnamic acid was caused by a continuous increase in enzyme activity of PAL and C4H after 6 h osmotic stress. Similarly, the accumulation of ferulic acid, *p*-coumaraldehyde, and sinapic acid was due to the enzyme activity of COMT, CCR, and F5H increased. The decrease of 4CL enzyme activity which is induced by decreased expression level of *4CL* (*MsG0580024209.01*) reduces substrate (ferulic acid, *p*-coumaric acid, and sinapic acid) consumption, and it eventually led to an increase in the accumulation of these substrates. Similarly, the increase of *p*-coumaraldehyde was due to that CCR activity increased continuously and CAD activity decreased at 6 h. It was worth noting that all these enzyme activities increased after the short-term (6 h or 1 day) of osmotic stress and the metabolite contents increased after long-term (4 days or 7 days) osmotic stress. The reason might be that osmotic stress induced a quick improvement of the lignin synthesis-related enzyme activities, thus resulting in metabolite accumulation in alfalfa leaves. However, in our previous study, while the enzyme activities increased, the accumulation of DAMs decreased in the stems of alfalfa [27]. We speculated that phenylalanine, the original substrate for the phenylpropane pathway, was sufficient for lignin synthesis in the leaves, resulting in a great increase in cinnamic acid content. The redundant substrates were then transported to the stem for lignin accumulation. The decreased metabolites suggested that the lignin synthesis might be restricted in the stem due to the priority supply of substrates to the leaf.

Lignin biosynthesis improved plant resistance to abiotic stress. Previous studies identified 28 DEGs belonging to eight families (*PALs*, *C4Hs*, *4CLs*, *COMTs*, *CCRs*, *CADs*, *PODs*, and *UGTs*), which took part in lignin synthesis under salt stress in *Sophora alopecuroides* [25]. In our research, we found 59 DEGs involved in the lignin synthesis. They belonged to 12 gene families, four (*HCT*, *CCRs*, *CADs*, *UGTs*) of which were all upregulated, indicating that upregulation of these genes may be one of the most crucial factors in lignin synthesis when alfalfa encounters osmotic stress. Some previous studies have found that drought stress increased CCR protein abundance and lignin content in *Leucaena* seedlings stems, suggesting that CCR-catalyzed lignin synthesis may be critical for drought stress tolerance of *Leucaena* [29]. *CmCAD2* was designated as an abiotic-stress-response gene that participates in lignin biosynthesis in oriental melons [30]. Two *CsHCT* genes were upregulated under the four stress treatments (cold, salt, drought, and MeJA stress) in tea plants [31]. In addition, all of the *CCRs*, *CADs*, and *UGTs* were also significantly upregulated in alfalfa stem tissue under osmotic stress [27], indicating that *CCRs*, *CADs*, and *UGTs* played an important role in both stems and leaves of alfalfa under osmotic stress. Meanwhile, *CCR* (*MsG0480018285.01*), *CADa* (*MsG0180005813.01*), and *CADb* (*MsG0280006638.01*) were hub genes identified by WGCNA analysis. Peroxidase (POD) is an enzyme that has multiple functions, and it participated in several diffident plant physiological processes, involving stress resistance, oxidation, and polymerization of lignin monomers after they were transported to the cell walls [32]. Under cold stress, the *POD* gene expression was upregulated in the roots and leaves of sweetpotato [32]. Transcript abundance of *POD* in leaves was obviously increased by salt shock in *Eutrema salsugineum* [33]. In this research, *CSE* (*MsG0180006172.01*) and *POD* (*MsG0380016745.01*) were hub genes for lignin synthesis. In our previous study, these two genes were also hub genes related to lignin biosynthesis in alfalfa stems under osmotic stress [27].

WGCNA analysis identified 30 edge genes, which were supposed to be very important to the regulation of lignin synthesis under osmotic stress. The edge genes included *MYB*, *WRKY*, and genes encoded ubiquitin-specific protease. *MYB15* was the homologous gene of *AtMYB15*, which played an important role in lignin synthesis in effector-triggered immunity [34]. Among homologous genes of edge genes, *AtMYB111*, *AtMYB113*, and *AtMYBD* took part in the regulation of anthocyanin and flavonol in phenylpropane metabolism [35, 36]. Notably, *UBP7*, as the homologous genes of *AtUBP7*, encoded a ubiquitin-specific protease associated with all five hub genes in leaves. We speculated that lignin biosynthesis was related to ubiquitination under osmotic stress. These results indicated that the lignin synthesis was regulated in multilevel under osmotic stress, including transcription and post-translational modification of proteins.

GO analysis revealed that the “abscisic acid-activated signaling pathway” and “ethylene-activated signaling pathway” were enriched. To test the influence of ABA and ethylene on lignin biosynthesis, we measured the lignin accumulations under ABA/ACC treatment. Lignin content significantly increased by ABA or mannitol (MAN) treatment alone and the combination of MAN + ABA treatment. However, the lignin content was significantly reduced by ABA inhibitor (FLU). These results indicated that ABA signal molecules positively regulated lignin accumulation, and it was consistent with previous reports in *Arabidopsis thaliana* [37], apple [6], melon [38], and poplar [39]. However, another study suggested that the lignin accumulation in leaves was not affected by exogenous ABA in maize [40]. We suspected that the effect of ABA on lignin accumulation was specific in different species. Although there have been many studies on the relationship between lignin synthesis and ethylene, studies on lignin regulation by ethylene under abiotic stress remain unclear. In this research, under osmotic stress, the lignin biosynthesis of alfalfa leaves was inhibited by ACC, and it was relieved by AgNO_3_, an ethylene inhibitor. We speculate that osmotic stress may change the ethylene signaling and other metabolites, which have antagonistic or synergistic effects, to regulate the biosynthesis of lignin negatively. The influence of ABA and ethylene on lignin biosynthesis under osmotic stress is consistent with the results of our previous studies on stem tissue [27].

## Conclusion

In summary, a combination of physiological, metabolomic, and transcriptomic analyses was performed to understand the lignin synthesis under osmotic stress in the leaves of alfalfa. Lignin content increased gradually under osmotic stress. The increase in enzyme activity promoted the accumulation of 5 metabolites. We identified 5 hub genes and constructed a co-expression network of lignin biosynthesis based on WGCNA analysis. In addition, ABA regulated lignin content positively, and ethylene regulated lignin content negatively under osmotic stress. These conclusions help us get a better understanding of lignin synthesis and regulation mechanism in alfalfa under osmotic stress, and provide new insight into the improvements of abiotic tolerance and quality of alfalfa. However, the function of the identified key genes needs to be further verified, and how ABA and ethylene regulate lignin needs to be further studied.

## Materials and methods

### Plant growth and treatment

We used Alfalfa (*M. sativa* L. cv. Zhongmu No.1) as plant material. The seeds were surface-sterilized in 75% ethanol for 10 min, and rinsed five times with sterilized water, then germinated in a petri dish 1/2 Murashige and Skoog (MS) medium with 1% sucrose with 40 seeds per dish. After two days of dark cultured at 4 ℃, continued dark culture at 25 ℃. 7 days old seedlings were transplanted into customized hydroponic pots (30 cm×15 cm) with modified Hoagland. Seedlings at 40 days old were treated with 100 mM mannitol for 6 h, 1 d, 4 d, and 7 d respectively, then the samples of leaf tissues were immediately frozen in liquid nitrogen and stored at -80 °C for metabolomic and transcriptomic analysis. All experiments were repeated three times with three biological replicates.

### Measurement of physiological indexes

#### Osmotic potential

Osmotic potential was determined by vapour pressure osmometer (Vapro 5600, Westcor, USA) using a standard 10 µl chamber. Solute concentration was converted to an osmotic potential (*Ψπ*) using the following formula: *Ψπ* = - *RTC*_*s*_, where *R* is a gas constant, *T* is temperature in Kelvin and *C*_*s*_ is the solute concentration [41].

#### Relative water content

The relative water content (RWC) was analyzed by the following formula: RWC = [(FW-DW)/(TW-DW)] × 100, where FW means fresh weight, DW means dry weight, and TW means turgid weight [42].

#### Lignin content

The total lignin content was determined using acetyl bromide method [43].

#### Enzyme activity

The enzyme activities were measured by the enzyme activity detection Kit (Solarbio, Beijing, China). Those enzymes include peroxidase (POD), laccase (LAC), 4–coumaric acid: CoA ligase (4CL), Cinnamate 4-hydroxylase (C4H), cinnamyl alcohol dehydrogenase (CAD), and phenylalanine ammonia-lyase (PAL). Caffeoyl shikimate esterase (CSE), hydroxycinnamoyltransferase (HCT), uridine diphosphate glycosyltransferases (UGT), caffeic acid O-methyltransferase (COMT), caffeoyl-CoA 3-O-methyltransferase (CCoAOMT), cinnamoyl-CoA reductase (CCR), ferulate 5-hydroxylase (F5H) were measured by ELISA assay Kit (Jingmei, Jiangsu, China).

### Transcriptomic sequencing and analysis

Total RNA was extracted using TRIzol methods, and cDNA library construction and sequencing were performed by Metware Biotechnology Co., Ltd. (Wuhan, China). Each group of RNA-sequenced samples included three biological replicates. Raw reads were filtered using fastp v0.19.3 [44]. Clean reads were aligned to the ‘Zhongmu No.1’ alfalfa genome using HISAT2 [45, 46]. Differential expression analysis between samples was performed using DESeq2 software, resulting in differentially expressed genes with the criteria of|log2Fold Change| ≥ 1 and a corrected *P* value < 0.05. The DEGs were used for Gene Ontology (GO) and Kyoto Encyclopedia of Genes and Genomes (KEGG) analyses [47].

### Metabolite extraction and quantification

The samples were extracted with 70% methanolic aqueous, and the extracts were analyzed using a UPLC-MS/MS system. Metabolites with|log_2_ fold change| > 1 and *P* < 0.05 were identified as different accumulation metabolites (DAMs).

### Weighted gene coexpression network analysis

The R package (v1.70) was used for weighted gene co-expression network analysis (WGCNA). The gene expression modules were classified with all the genes, and trait parameters were accumulations of lignin and metabolites. The hub genes were the structural genes involved in lignin biosynthesis, and they were selected from the most correlated modules. Cytoscape 3.9.1 was used to construct a coexpression regulatory network.

### Real-time quantitative PCR analysis

Four hub genes were used for real-time quantitative PCR (RT-qPCR). Primers which were designed by Primer Premier 5.0 were listed in Table S1. ChamQ SYBR qPCR Master Mix (Vazyme, Nanjing, China) was used to investigate the relative expression, which was analyzed using the 2^–ΔΔCt^ method. There were three biological replicates and three technical replicates in each experiment.

### ABA or ACC treatment

To investigate the influence of ABA and ethylene on lignin contents under osmotic stress, forty days old alfalfa plants were treated with different reagents. For ABA treatment, 100 µM solution was sprayed on the alfalfa leaf; for ACC treatment, 100 µM ACC was performed by adding the chemicals to the nutrient solution; for osmotic stress, 100 mM mannitol was added to the nutrient solution; for MAN + ABA or MAN + ACC treatment, after ABA or ACC treatment, the plants were transferred to mannitol treated nutrient solution; for MAN + Flu (fluridone, ABA inhibitor, 25 µM ) and MAN + AgNO_3_ (ethylene inhibitor, 25 µM) treatment, inhibitors were added to the nutrient solution 10 h before treatment; for MAN + ABA + Flu or MAN + ACC + AgNO_3_ treatment, the inhibitor was applied to the nutrient solution for 10 h and then treated with ABA or ACC and 100 mM mannitol was immediately added to the nutrient solution. Untreated plants were taken as CK. After 4 days of treatments, the leaf tissues were collected for lignin content analysis.

### Statistical analysis

All statistical analyses were conducted by one-way analysis of variance (ANOVA) with Duncan’s multiple range method test at the 5% level using SPSS 26.0.

### Electronic supplementary material

Below is the link to the electronic supplementary material.

Supplementary Material 1

## Data Availability

The raw sequencing data in this study were submitted to the National Center for Biotechnology Information Sequence Read Archive (SRA) (https://dataview.ncbi.nlm.nih.gov/object/PRJNA1019078) with SRA accession number PRJNA1019078.

## Declarations

### Declaration of plant material

All of the plant material is owned by the authors and no permissions are required.
